# Biomarkers of axonal damage to favor early diagnosis in variant transthyretin amyloidosis (A-ATTRv)

**DOI:** 10.1038/s41598-023-50212-2

**Published:** 2024-01-05

**Authors:** Juan González-Moreno, Álvaro Gragera-Martínez, Adrián Rodríguez, Cristina Borrachero-Garro, Sandra García-Garrido, Carles Barceló, Ana Manovel-Sánchez, Maria Antonia Ribot-Sansó, Lesly Ibargüen-González, Rosa Gomila, Francisco Muñoz-Beamud, Inés Losada-López, Eugenia Cisneros-Barroso

**Affiliations:** 1 Balearic Research Group in Genetic Cardiopathies, Sudden Death and TTR Amyloidosis, Health Research Institute of the Balearic Islands (IdISBa), Palma, Balearic Islands Spain; 2 Clinical Analysis Department, Genetic Unit, Hospital Universitario Juan Ramón Jiménez, Huelva, Spain; 3 Internal Medicine Department, Hospital Universitario Juan Ramón Jiménez, Huelva, Spain; 4 Internal Medicine Department, Hospital Universitario Son Llàtzer, Crta Manacor Km 4, 07198 Palma, Balearic Islands Spain; 5 Translational Pancreatic Cancer Oncogenesis Group, Health Research Institute of the Balearic Islands (IdISBa), Palma, Spain; 6 Department of Chemistry, Universitat de les Illes Balears, Crta de Valldemossa Km 7.5, 07122 Palma de Mallorca, Baleares Spain; 7 Cardiology Department, Hospital Universitario Juan Ramón Jiménez, Huelva, Spain; 8 Multidisciplinary ATTR Unit, Hospital Universitario Juan Ramón Jiménez, Huelva, Spain; 9 Servicio de Medicina Interna, Hospital Universitario Son Llàtzer, Crta Manacor Km 4, 07198 Palma, Spain

**Keywords:** Biomarkers, Neurological disorders

## Abstract

Early identification of ATTRv amyloidosis disease onset is still often delayed due to the lack of validated biomarkers of this disease. Light chain neurofilament (NfL) have shown promising results in early diagnosis in this disease, but data is still needed, including with alternative measuring methods. Our aim was to study the levels of NfL measured by ELISA. Furthermore, interstitial matrix metalloproteinase type 1 (MMP-1) serum levels were measured as a potential new biomarker in ATTRv. Serum NfL and MMP-1 were measured using ELISA assays in 90 participants (29 ATTR-V30M patients, 31 asymptomatic V30M-*TTR* variant carriers and 30 healthy controls). Median NfL levels among ATTRv amyloidosis patients were significantly higher (116 pg/mL vs 0 pg/mL in both comparison groups). The AUC comparing ATTRv amyloidosis patients and asymptomatic carriers was 0.90 and the NfL concentration of 93.55 pg/mL yielded a sensitivity of 79% and a specificity of 87%. NfL levels had a significant positive correlation with NIS values among patients. We found a negative significant correlation between eGFR and NfL levels. Finally, MMP1 levels were not different between groups. Evidence of NfL use for early diagnosis of ATTR-PN amyloidosis is growing. ELISA seems a reliable and available technique for it quantification. Decreased GFR could influence NfL plasma levels.

## Introduction

Transthyretin variant amyloidosis (A-ATTRv) is a debilitating inherited disease that can be fatal without effective treatment^1^. It is caused by pathogenic variants in the transthyretin (TTR) gene, which is inherited in an autosomal dominant pattern^1^. V30M (p.V50M) is the most common *TTR* variant and is associated with ATTR amyloidosis-related polyneuropathy (A-ATTRv-PN)^2^. The most common clinical manifestations are dysautonomic and sensory symptoms due to small fibre polyneuropathy, followed by a rapidly progressive axonal sensorimotor polyneuropathy^3^.

As A-ATTRv-PN is a rapidly progressive and irreversible disease, early diagnosis is crucial^4^. Currently, diagnosis of the disease is primarily based on clinical data, particularly the presence of polyneuropathy, confirmed by neurophysiological test, and genetic testing to confirm the presence of pathogenic *TTR* variants^5^. However, early diagnosis, monitoring of disease progression and response to treatment can be challenging. Quality of life scales, nerve conduction studies, echocardiography and cardiac biomarkers are commonly used to monitor patients, but these methods have limitations as they are often used to assess damage that has already occurred.

Biomarkers reflect the pathophysiology of the disease and are therefore highly specific and useful for disease screening, diagnosis and monitoring^6^. While biomarkers such as NTproBNP or troponins are widely used in A-ATTR-related cardiomyopathy (A-ATTR-CM) and are already recommended in clinical practice guidelines^7^, there is a lack of specific biomarkers in A-ATTR-PN. A number of biomarkers have been postulated, but none has yet reached clinical practice. The most promising biomarker is neurofilament light chain (NfL)^8,9^. Evidence for its potential utility in diagnosis and follow-up is growing rapidly. A recent study evaluated changes in protein levels in patients with ATTRv amyloidosis with polyneuropathy treated with patisiran and found that NfL was the most significantly altered protein^8^. The proteome of patisiran-treated patients was observed to trend towards that of healthy controls at 18 months. Healthy controls had significantly lower levels of NfL compared to patients with ATTRv amyloidosis with polyneuropathy, and NfL levels decreased with patisiran treatment but increased with placebo. Improvement in neuropathy scores was significantly correlated with reduced NfL levels^8^.

In addition to A-ATTRv-PN, NfLs have shown a potential role as biomarkers of neuroaxonal injury in several central nervous system disorders, including Alzheimer's disease, multiple sclerosis and Huntington's disease, as well as peripheral nervous system disorders such as Guillain-Barré syndrome, Charcot-Marie-Tooth disease and chronic inflammatory demyelinating neuropathy^10–13^.

Other potential biomarkers include interstitial matrix metalloproteinases (MMPs), which are produced as a product of axonal degradation. In one study, MMP-14 levels were elevated in the plasma of A-ATTRv patients and correlated with disease progression^14^. MMP-9 has also been shown to be elevated in A-ATTR-PN^15^. As inflammation has been implicated in the pathophysiology of A-ATTR-PN, measuring MMP expression has been postulated as a potential target^14^.

The aim of this study is to analyse the potential role of NfL levels in the early diagnosis of A-ATTRv in a cohort of A-ATTR-V30M patients. As an exploratory parameter, we also analysed interstitial matrix metalloproteinase 1 (MMP1) and its potential role in the diagnosis of A-ATTRv.

## Methods

We performed a single-centre, prospective, cross-sectional and analytical study to establish an association between biomarkers and clinical expression of A-ATTRv-PN.

### Study population

A-ATTR-V30M patients, asymptomatic V30M-*TTR* mutation carriers and healthy controls were recruited at the outpatient clinics of the Hospital Universitario Son Llàtzer between November 2020 and April 2021. Patients with other potential cause of polyneuropathy or neurological disease were excluded. The study was approved by the Comité de Ética de las Islas Baleares. All individuals agreed to participate in the study by signing an informed consent form. The study was conducted in accordance with the Declaration of Helsinki.

### Clinical evaluation

Clinical assessment included: demographics, current A-ATTRv treatment, complete clinical examination and NIS. Clinical phenotype was defined as previously: early-onset if ATTRv disease started before 50 years of age and late-onset if it started after 50 years of age. Functional status was assessed with the Polyneuropathy Disability Score (PND) and the Familial Amyloid Polyneuropathy Scale (FAP) as described elsewhere^16^. Laboratory measurements included: serum transthyretin, albumin and total protein levels. Serum transthyretin levels were quantified by nephelometry (Beckman coulter) according to manufacturer instructions.

### Sample collection and processing

Serum samples were collected from A-ATTRv patients, asymptomatic *TTR* mutation carriers and healthy controls. Samples were collected using standard venipuncture techniques and processed within two hours of collection. Immediately after collection, the samples were centrifuged at 2,000 g for 10 min to obtain the serum fraction, which was then aliquoted into cryovials. The aliquoted serum samples were stored at −80 °C until further analysis.

Serum NfL levels were measured using the Uman Diagnostics NF-Light^®^ Serum ELISA assay. This is an enzymatic immunoassay using two highly specific, non-competing monoclonal antibodies. The capture antibody is coated onto a solid surface and binds to NfL molecules. The detection antibody is biotin conjugated and the addition of HRP-conjugated streptavidin allows quantitative determination by enzymatic conversion of a colourless substrate (TMB) to a coloured product. Serum samples were thawed at room temperature and gently mixed prior to testing. The Quanterix NfL ELISA kit was used to quantify NfL levels in the serum samples according to the manufacturer's instructions. The assay allows the detection and quantification of NfL in the samples. The samples, controls and standards were added to the ELISA plate and the assay was performed according to the protocol provided. Optical density readings were obtained using a microplate reader set at the appropriate wavelength, and the corresponding NfL concentrations were calculated from the absorbance in each sample using a standard curve generated from the standards provided. This assay has a low limit of detection (LLoD) of 33 pg/mL and a variant coefficient (%) between < 5% inter and < 10% intra-precision respectively.

Serum MMP-1 levels were measured using the RayBio^®^ Human MMP-1 ELISA Kit from RayBiotech^®^. This is an in vitro enzyme-linked immunosorbent assay for the quantitative measurement of human MMP-1 pro and active forms in serum. In this assay, an antibody specific for human MMP-1 is coated in each well and a second biotinylated anti-human MMP-1 antibody is added. The ELISA assay is performed according to the manufacturer's instructions, colour change is measured and MMP-1 levels are calculated using a standard curve generated from supplied standards.

Both the NfL and MMP-1 ELISAs were performed in an automated microplate reader, Triturus, Grifols Diagnosis^®^.

### Statistical analysis

After assessing the normality of the data, descriptive statistics including means, standard deviations (SD), medians and interquartile ranges (IQR) were calculated accordingly for continuous variables, while frequencies and percentages were used for categorical variables. Non-parametric Kruskal–Wallis tests were used to compare NfL levels between the three groups, followed by the Bonferroni post-hoc test. Receiver operating characteristic (ROC) curve analysis was performed to assess the ability of NfL to discriminate between groups. The Youden index was used to estimate the best cut-off point. Non-parametric correlation analyses were performed to assess the relationships between NfL levels and other variables of interest, and the Rho Spearman coefficient was reported. Linear regression analysis was used to determine the independent association of NfL with glomerular filtration rate (eGFR), adjusting for potential confounders.

SPSS v23 and GraphPad were used as statistical software. A p < 0.05 was considered statistically significant.

### Ethics approval

Ethical approval was granted by the Ethics Committee of the Balearic Islands and the Research Commission of Hospital Universitario Son Llàtzer. Decission number: IB 4491/21 PI.

## Results

### Demographic and clinical characteristics

Serum samples were collected from 29 A-ATTR-V30M patients, 31 asymptomatic carriers of the V30M-*TTR* variant and 30 healthy controls. The main characteristics of the enrolled subjects are summarised in Table 1. All but 4 A-ATTRv patients had neurological involvement, most of them with late disease onset (20 patients, 80.0%). Most patients were in an early stage of the disease (72.4% were in FAP 0-I), with a global median NIS of 8. Twenty-two patients (75.9%) were receiving A-ATTRv-specific treatments at the time of the study. Among the untreated patients, 4 patients (66.6%) had a cardiological phenotype with no specific treatment option at the time of the study and 2 patients (33.3%) had advanced disease (FAP III).

**Table 1:** Main characteristics of the study cohort.

	A-ATTR-V30M patients (n = 29)	V30M asymptomatic carriers (n = 31)	Healthy controls (n = 30)
Median age (range)	69 years (29–86)	48 years (21–81)	43 years (20–73)
Females (%)	11 (37.9%)	18 (58.1%)	18 (60%)
mean eGFR (SD)	74.3 mL/min (21.2)	92.6 mL/min (16.7)	
Mean Total proteins (SD)	7.2 g/dL(0.5)	7.2 g/dL (0.4)	
Mean serum Albumin (SD)	4.3 g/dL (0.3)	4.5 g/dL (0.3)	
Median NIS (range)	8 (0–96)		
PND			
0	4 (13.8%)		
I	17 (58.6%)		
III	6 (20.7%)		
IV	2 (6.9%)		
FAP			
0	4 (13.8%)		
1	17 (58.6%)		
2	6 (20.7%)		
3	2 (6.9%)		
Clinical phenotype			
Neurological early onset	5 (17.2%)		
Neurological late onset	20 (69.0%)		
Cardiological	4 (13.8%)		
Treatment			
Tafamidis	8 (31.0%)		
Patisiran	12 (41.4%)		
Inotersen	2 (6.9%)		
No treatment	6 (20.7%)		

A-ATTRv patients were significantly older than both asymptomatic carriers (mean age 67 vs 50 years) and healthy controls (66.9 vs 42.8 years) (p < 0.001 in both cases), and there were fewer females in the former group (37.9% vs 58.1% vs 60%). A-ATTRv patients also have a lower mean estimated eGFR than asymptomatic carriers (mean eGFR 74.3 vs 92.6; p < 0.001).

### NfL levels were higher among patients

**Figure 1:**
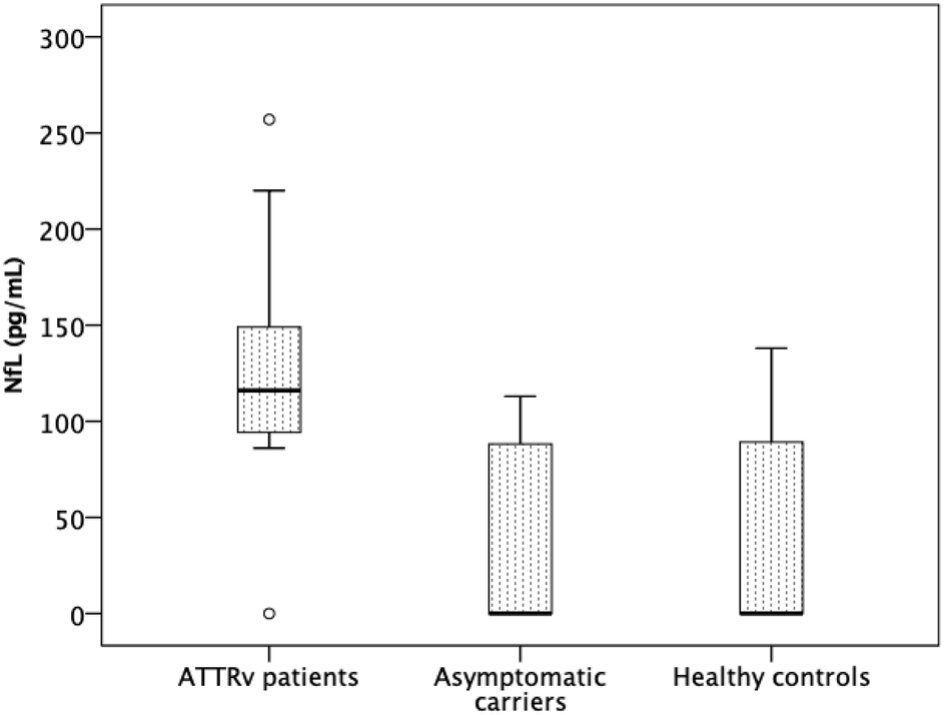
Serum neurofilament light chain concentration in A-ATTR-V30M patients, asymptomatic V30M TTR variant carriers and healthy controls.

Median NfL levels were 116 pg/mL (IQR 93–150 pg/mL) in A_ATTRv patients, < 33 pg/mL (IQR < 33–90 pg/mL) in asymptomatic variant carriers and < 33 pg/mL (IQR < 33–90 pg/mL) in healthy controls (Fig. 1). These differences were statistically significant between A-ATTRv patients and both asymptomatic carriers (p < 0.001) and healthy controls (p < 0.001). No statistical differences in NfL levels were observed between asymptomatic carriers and healthy controls.

**Figure 2:**
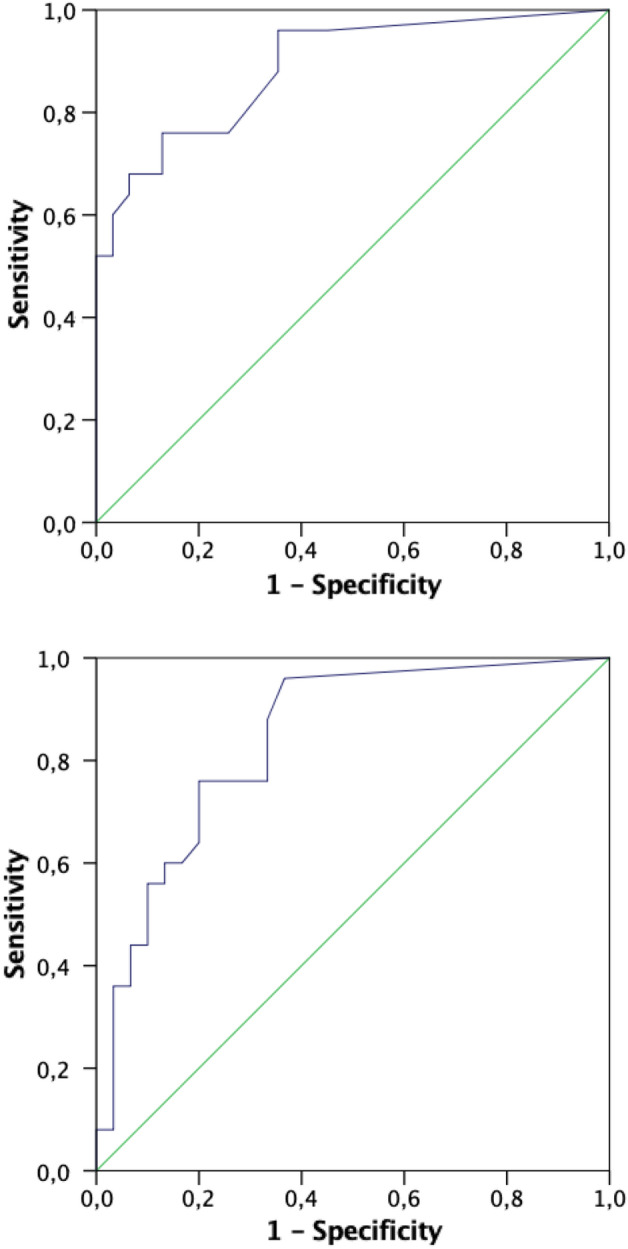
(**A**) ROC curve analysis of NfL serum levels in A-ATTR-V30M patients vs. asymptomatic V30M-TTR variant carriers. (**B**) ROC curve analysis of NfL serum levels in A-ATTR-V30M patients vs. healthy controls.

ROC curve analysis was used to compare the ability of NfL to discriminate between A-ATTRv patients and both asymptomatic carriers (Fig. 2A) and healthy controls (Fig. 2B). The AUC comparing A-ATTRv patients and asymptomatic carriers was 0.90 (CI 95% 0.83–0.98; p < 0.001) and the NfL concentration of 93.55 pg/mL gave a sensitivity of 79% and a specificity of 87%. The AUC comparing A-ATTRv patients and healthy controls was 0.86 (CI 95% 0.77–0.95; p < 0.001) and the NfL concentration of 92.6 pg/mL gave a sensitivity of 79% and a specificity of 80%.

### NfL correlates with disease severity

**Figure 3:**
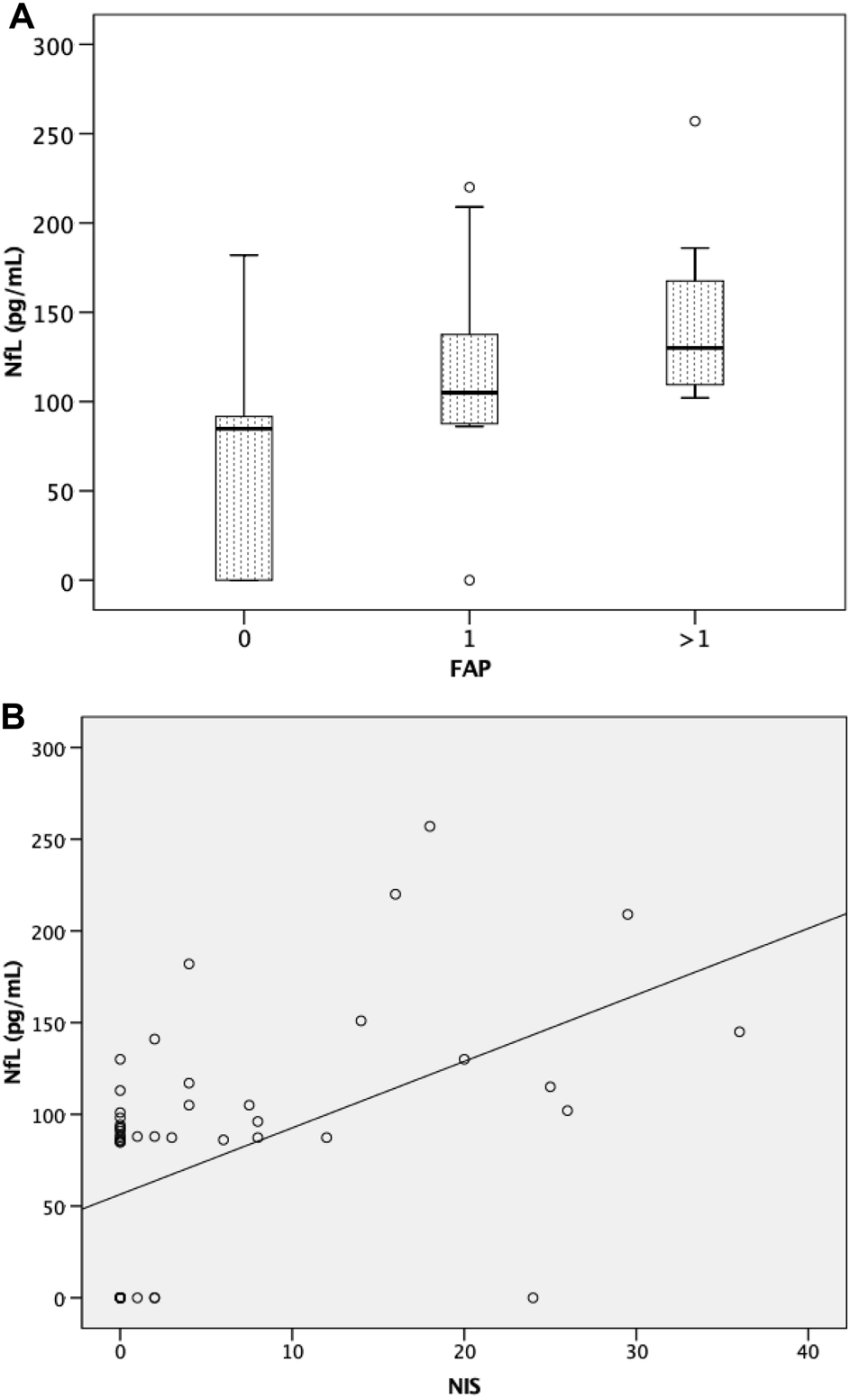
(**A**) Serum neurofilament light chain concentration at different disease stages in A-ATTR-V30M patients. (**B**) Correlation of serum neurofilament light chain concentration and NIS values in A-ATTR-V30M patients.

While NfL levels were significantly higher in patients with FAP stage 1 than in asymptomatic carriers or patients with cardiac phenotype FAP 0 (p < 0.001), no differences were observed between FAP 1 patients and those with more advanced disease (FAP > 1). On the other hand, NfL levels have a significant positive correlation with NIS levels in patients with Rho = 0.630 (p < 0.001) (Fig. 3).

### NfL correlates with age and estimated glomerular filtration rate (eGFR)

We found a significant correlation between age and NfL levels (Rho = 0.365; p < 0.001). We also found a negative significant correlation between eGFR and NfL levels (Rho = −0.388; p < 0.001). No significant correlation was found between NfL and mBMI nor TTR serum levels, and no differences in NfL levels were observed between the sexes. After adjustment for disease status, severity and age, linear regression showed that eGFR was independently associated with NfL (p < 0.001). Furthermore, after excluding patients with an eGFR below 60 mL/min, significant differences in NfL were still observed between groups.

### MMP1 levels were not different between groups.

Median MMP1 levels were 10,735 pg/mL (IQR 6489–15,927) in A-ATTRv patients, 7187 pg/mL (IQR 4248–13,725) in asymptomatic variant carriers and 7228 pg/mL (IQR 3246–11,755) in healthy controls. These differences between groups were not statistically significant. MMP1 levels did not differ between groups when compared by disease severity.

## Discussion

Our study confirms the evidence for the usefulness of NfL for the early diagnosis of A-ATTR-V30M-related polyneuropathy. Furthermore, we have shown that ELISA could also be a reliable method for NfL quantification. Finally, we also found that renal function, in addition to age, should be considered when analysing NfL levels.

Serum NfL levels are known to be elevated in the presence of axonal degeneration. In healthy controls, insignificant levels of NfL have been shown^17,18^. Thus, elevated NfL levels may represent a surrogate biomarker for disease onset in A-ATTRv-related neuropathy in the absence of other possible aetiologies of neuropathy, as NfL may be elevated in other peripheral and central nervous system disorders. Indeed, these differences have been shown even in the very early stages of the disease^17^, as we have also shown. A-ATTRv related polyneuropathy usually starts with small fibre involvement^3^. As NfL have been shown to be elevated even in patients without large fibre neuropathy, they are an excellent biomarker for early diagnosis. The gold standard for the diagnosis of small fibre neuropathy is skin biopsy to assess intraepidermal nerve fibre density (IENFD)^19^. Unlike invasive testing, this technique is not universally available, so NfL could provide a non-invasive and more available test for early detection of small fibre involvement. Previous studies have also shown an association between NfL and disease severity. While we have shown significant differences between NfL levels in asymptomatic carriers and early stage disease (FAP = 1), we have not shown differences between FAP = 1 and FAP > 1 patients. On the other hand, we found a strong correlation between NfL levels and NIS, suggesting an increase in NfL levels in more advanced stages of the disease. As our cohort was mainly in the early stages of the disease, the differences between FAP stages were probably not significant. However, previous studies have shown differences in NfL levels according to disease stage, using either FAP or PND stages^10,17,18^. Loser et al. also described a strong correlation between NfL and NIS, ESC in the feet and NCS sensory scores^9^. However, as symptomatic patients are usually treated with specific A-ATTR treatments, disease progression may be halted and thus NfL levels may be reduced. Some previous work has shown how treatment can reduce NfL levels and therefore how these biomarkers could be used to monitor treatment response^8,9,20^.

As previously reported, we found a significant correlation between NfL levels and age^17^. However, NfL differences between A-ATTRv patients and both healthy carriers and controls have been shown to be largely independent of age, as we also found^17^. On the other hand, we also showed a strong negative correlation between NfL levels and eGFR. This has been observed previously^21,22^. It is not known why reduced eGFR might alter NfL levels. As NfL are too large (62 kDa) to be filtered by the glomerulus, the presence of lower molecular weight fragments of NfL recognised by the antibodies used in NfL assays could explain the influence of eGFR on NfL plasma levels^21^. Although it is possible that an age-related decline of eGFR rather than a disease effect on kidney function could be the reason of lower eGFR among patients compared to asymptomatic carriers^23^, the influence of eGFR on NFL levels was shown to be age independent.

Although TTR circulating levels has been suggested to be associated with onset of symptoms in A-ATTRv with polyneuropathy and low serum TTR levels have been thought to be associated with a poor prognosis in patients with A-ATTR cardiomyopathy^24,25^, we found no correlation between TTR and NfL plamsa levels in our cohort.

Mean NfL values were higher in all groups of our cohort compared to previous studies^9,17^. The vast majority of published studies use Simoa^®^ to measure NfL^17,20^. However, this technique is expensive and not widely available. Instead, we have used ELISA, with this first test having a relatively high limit of detection (33 pg/mL). Recently, we are using second generation ELISA, The Uman Diagnostics NF-Light^®^ ELISA SERUM assay, Quanterix^®^, that has a very low detection limit (0.4 pg/mL) as other groups have done^26^. ELISA appears to be a reliable and cost-effective technique for NfL quantification, but absolute NfL levels may differ^22^. In the last years, Simple Plex assay for the detection of human NfL allow its quantification with a reasonable detection limit (2.7–10,290 pg/mL), with a low cross reactivity and cheaper than Simoa^®^. This could become the third candidate con NfL measurement^27^.

The exact threshold of NfL levels to discriminate asymptomatic carriers from A-ATTRv patients has not been established. Previous studies have suggested thresholds ranging from 10.6 pg/ml to 37 pg/ml^8,9,17,28^. It is noteworthy that median NfL levels in our cohort is < 33 pg/ml. However, it appears that rather than using a single NfL cut-off, individual cut-off percentiles and z-scores based on the reference healthy control population should be used^9^. In one study, a z-score cut-off of 1.45 could discriminate the two groups with a sensitivity of 85.7% and a specificity of 100%^9^.

We only included patients with V30M, the main variant presenting with A-ATTR-PN. However, other studies have shown the potential benefit of NfL in non-V30M patients^18,23^. Furthermore, the use of NfL has not been limited to A-ATTR amyloidosis, but also to other amyloidoses such as AL^10^ and other neurological diseases such as multiple sclerosis, Charcot-Marie-Tooth, chronic inflammatory demyelinating polyneuropathy or Alzheimer's disease^11–13^.

Previous studies have suggested a potential role for matrix metalloproteases (MMPs), particularly MMP-14, in A-ATTRv with polyneuropathy^14^. In our study, MMP1 levels did not differ between groups. Due to differences in location and substrate specificity, it is likely that not all MMPs may play a role in A-ATTRv pathogenesis and thus have a role as biomarkers.

Soluble non-native TTR conformations (NNTTR) have also been proposed as a biomarker for A-ATTR amyloidosis^26^. Although less studied, NNTTR appears to be specific for A-ATTR-related neuropathy, as NNTR were not found in patients with other peripheral neuropathies^26^. Furthermore, NNTR may be a biomarker for treatment response, as higher levels of NNTR were associated with poorer treatment response^26^.

Finally, a possible role of systemic inflammation has been proposed as a potential pathologic pathway in A-ATTR. Thus, some inflammatory markers, as IFN-alpha or IFN-gamma, has been shown to be overexpressed in A-ATTR patients compared to healthy controls. However, more evidence is still needed to confirm these observations^29,30^.

### Limitations

Our study has several limitations. Our study was performed using first generation ELISA for NfL measurement, with an elevated limit of detection (33 pg/mL). Despite this, clear differences between groups were detected. Our cohort was relatively small and heterogeneous to find other correlations of NfL. However, it was sufficient to find differences between carriers and patients, allowing us to use NfL for early diagnosis. The cross-sectional design of our study did not allow us to find differences in NfL levels after treatment initiation. A-ATTRv patients in our cohort were older than both asymptomatic carriers and controls. However, we still showed differences in NfL levels between groups independent of age. Our study included only V30M Majorcan patients, so the results cannot be extrapolated to other populations.

## Conclusions

In conclusion, the evidence for the use of NfL in the early diagnosis of A-ATTR-PN is growing. ELISA appears to be a reliable and available technique for its quantification. Decreased eGFR may influence NfL circulating levels.

## Data Availability

The datasets generated and/or analysed as part of the current study are not publicly available, as patient privacy issues are involved in this study and there are ongoing follow-up studies, and the data are currently confidential but are available from the corresponding author on reasonable request.
